# Consumption of sugary soft drinks among children and adolescents in Germany. Results of the cross-sectional KiGGS Wave 2 study and trends

**DOI:** 10.17886/RKI-GBE-2018-024

**Published:** 2018-03-15

**Authors:** Gert B.M. Mensink, Anja Schienkiewitz, Martina Rabenberg, Anja Borrmann, Almut Richter, Marjolein Haftenberger

**Affiliations:** Robert Koch Institute, Berlin, Department of Epidemiology and Health Monitoring

**Keywords:** SOFT DRINKS, BEVERAGE CONSUMPTION, HEALTH SURVEY, CHILDREN AND ADOLESCENTS, KIGGS

## Abstract

Consuming large amounts of sugary beverages has been related to developing obesity, diabetes mellitus type II and other chronic diseases. KiGGS Wave 2 (2014-2017) provides data on the consumption of sugary soft drinks in the 3-17 year age group in Germany. Overall, 13.7% of girls and 17.6% of boys consume one to three times a day sugary soft drinks and 3.3% of girls and 4.7% of boys four or more times. Consumption frequency increases with age and is higher among children and adolescents with low socioeconomic status (SES) than for those of the same age with high SES. The share of adolescents who drink sugary soft drinks daily has decreased since the KiGGS baseline study (2003-2006).

## Background

Constituting a risk factor for overweight and obesity, the consumption of sugary beverages has been in the scientific and political spotlight in recent years [1]. Many studies indicate a link between high levels of consumption of sugary soft drinks among children and adolescents and weight increase [2]. While drinking sufficient amounts of liquid is important, as far as this need is mainly met with sugary soft drinks, it can in the long term lead to weight gain. Comparably, large amounts of calories are ingested without a corresponding effect on satiety. If these extra calories are not counterbalanced in energy expenditure, this may cause overweight in the long term. Consuming large quantities of sugary soft drinks also increases the risk for diabetes mellitus type II [3]. Blood sugar levels increase rapidly after consumption and the body produces greater amounts of insulin. Larger fluctuations in blood sugar levels appear, which can damage the insuline producing cells in the pancreas in the long term. Furthermore, consuming sugary soft drinks stresses the teeth because both the sugar and acids often contained in soft drinks attack tooth enamel and promote caries [4]. Studies, moreover, indicate a link between consumption of sugary soft drinks and reduced bone density at adolescent age, potentially caused by the acids contained in, for example, cola beverages (such as phosphoric acid) [5]. The German Nutrition Society (DGE) therefore recommends drinking mainly water and other low-calory beverages to cover liquid needs [6].

Against this backdrop, it is worrying that the per capita consumption of soft drinks, most of which contain sugar, is relatively high in Germany [7, 8]. Current data provided by the Federal Ministry of Food and Agriculture (BMEL) show that the consumption of soft drinks has increased from 117 litres in 2008 to 126 litres per capita in 2013. Nonetheless, consumption levels have again dropped since 2013 and were down to 119 litres per capita in 2015 [7]. Between 2012 and 2016, per capita lemonade consumption dropped from 83 to 78 litres [8]. Surveys on individual beverage consumption patterns from recent years indicate the popularity of sugary soft drinks in Germany, in particular among children and adolescents [9]. Data of KiGGS Wave 2 (2014-2017) provide a current overview of childrens and adolescents consumption of soft drinks and an evaluation of the development of consumption levels since the KiGGS baseline study (2003-2006).

**KiGGS Wave 2**

Second follow-up to the German Health Interview and Examination Survey for Children and Adolescents

**Data owner:** Robert Koch Institute

**Aim:** Providing reliable information on health status, health-related behaviour, living conditions, protective and risk factors, and health care among children, adolescents and young adults living in Germany, with the possibility of trend and longitudinal analyses

**Study design**: Combined cross-sectional and cohort study

**Cross-sectional study in KiGGS Wave 2**

**Age range:** 0-17 years

**Population:** Children and adolescents with permanent residence in Germany

**Sampling:** Samples from official residency registries - randomly selected children and adolescents from the 167 cities and municipalities covered by the KiGGS baseline study

**Sample size:** 15,023 participants

**KiGGS cohort study in KiGGS Wave 2**

**Age range:** 10-31 years

**Sampling:** Re-invitation of everyone who took part in the KiGGS baseline study and who was willing to participate in a follow-up

**Sample size:** 10,853 participants

**KiGGS survey waves**

► KiGGS baseline study (2003-2006), examination and interview survey
► KiGGS Wave 1 (2009-2012), interview survey
► KiGGS Wave 2 (2014-2017), examination and interview survey

More information is available at www.kiggs-studie.de/english

## Indicator and methodology

KiGGS is part of the health monitoring system at the Robert Koch Institute. It includes repeated cross-sectional surveys that are representative for children and adolescents aged between 0 and 17 years in Germany (KiGGS cross-sectional study). After conducting the baseline interview and examination survey between 2003 and 2006, and KiGGS Wave 1 as an interview only survey between 2009 and 2012, KiGGS Wave 2 was conducted between 2014 and 2017, again as a combined interview and examination survey.

A detailed description of the methodology used in KiGGS Wave 2 can be found in New data for action. Data collection for KiGGS Wave 2 has been completed in issue S3/2017 as well as KiGGS Wave 2 cross-sectional study – participant acquisition, response rates and representativeness in issue 1/2018 of the Journal of Health Monitoring [10, 11].

Like in the baseline study and in the German Health Interview and Examination Survey for Adults (DEGS 1, 2008-2011), in KiGGS Wave 2 the consumption of selected food items was assessed with a food frequency questionnaire [12, 13]. The questionnaire also asked about beverage consumption ‘during the past four weeks’. For the 3-10 year age group, parents or legal guardians provided answers, whereas participants aged 11-17 years answered the questions themselves. The question about the frequency of soft drink consumption was: ‘How often during the past four weeks did your child/did you drink sugary soft drinks (such as cola, lemonade, ice tea, malt beer or energy drinks)? This does not include diet beverages.’ The possible answers were: ‘never’, ‘once per month’, ‘2-3 times per month’, ‘1-2 times per week’, ‘3-4 times per week’, ‘5-6 times per week’, ‘once per day’, ‘2 times per day’, ‘3 times per day’, ‘4-5 times per day’, ‘more than 5 times per day’. Frequencies of soft drink consumption were summarised into three categories for the analysis presented here: ‘less than once per day’, ‘one to three times per day’ and ‘four or more times per day’. The average portion size was determined by asking, ‘When your child/you drink sugary soft drinks, how much does your child/do you usually drink?’ The answers ranged from: ‘½ a glass (or less)’, ‘1 glass (200 ml)’, ‘2 glasses’, ‘3 glasses’ and ‘4 glasses (or more)’.

For a comparison with the baseline study, the data on consumption frequency was converted and multiplied ((consumption frequency per 28 days x portion size (g))/28 days) to calculate estimated mean daily amounts.

The analyses are based on the data provided by 12,978 children and adolescents (6,539 girls and 6,439 boys) aged 3-17 years with valid responses on the consumption of sugary soft drinks. The results are presented as prevalence (frequency) according to gender, age and socioeconomic status (SES) [14].

**Info box**

Soft drinks include lemonades, fizzy drinks, fruit spritzers and fruit juice drinks, these usually contain added sugar. The analyses include some further drinks such as malt beer, ice teas and energy drinks [9].

The calculations were conducted applying a weighting factor that corrects deviations from the German population within the sample with regard to age, gender, federal state, nationality as well as the distribution of parent levels of education (Microcensus 2013 [15]). The analyses also take the cluster design of the sample into account. This article reports prevalences with 95% confidence intervals (95% CI). Differences between groups are interpreted as statistically significant if the corresponding confidence intervals do not overlap.

## Results and discussion

Overall, 13.7% of girls and 17.6% of boys drink sugary soft drinks one to three times per day and 3.3% of girls and 4.7% of boys four or more times per day. Daily consumption is slightly higher for boys than for girls of the same age. Consumption frequency rises with age and is highest among 14 to 17 year olds (girls 21.1%, boys 32.2%; Table 1).

Children and adolescents with low SES drink sugary soft drinks significantly more often compared to their peers with medium SES and the latter, in turn, more than those with high SES (Table 1). This difference is statistically significant. The vast majority of children and adolescents in the 3 to 17 age group drinks less than one sugary soft drink per day. Around 16.3% of girls and 12.7% of boys never drink sugary soft drinks (data not shown). The share of children and adolescents who drink sugary soft drinks every day has decreased since the KiGGS baseline study (2003-2006). In the baseline study, 28.2% of girls and 34.0% of boys reported drinking sugary soft drinks at least once per day [16]. This figure has now dropped to 16.9% for girls and 22.2% for boys. This is a desirable development which can also be seen in some other countries. Between 2003 and 2004, nearly 80% of child respondents in the US NHANES survey (National Health and Nutrition Examination Survey) reported drinking sugar-sweetened beverages on a particular day. In the 2013-2014 survey this figure had dropped to 61% of children [17].

**Figure 1:**
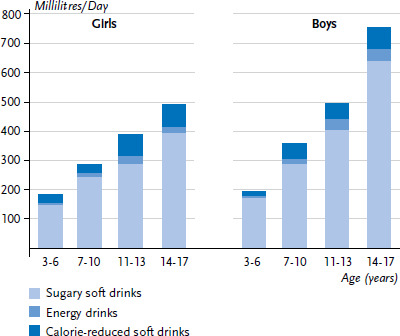
Calculated mean consumption of sugary soft drinks (millilitres/day) for participants of the KiGGS baseline study according to gender and age (n=6,847 girls, n=7,103 boys) Source: KiGGS baseline study (2003-2006)

**Figure 2:**
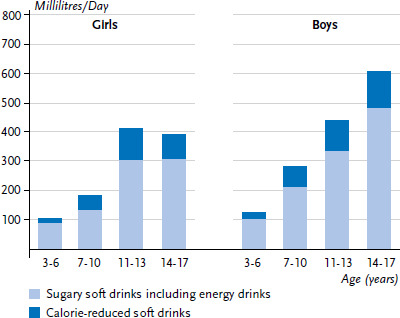
Calculated mean consumption of sugary soft drinks (millilitres/day) for participants of KiGGS Wave 2 according to gender and age (n=6,516 girls, n=6,424 boys) Source: KiGGS Wave 2 (2014-2017)

When comparing consumption frequencies between the KiGGS baseline study and KiGGS Wave 2, it must be considered that the corresponding questions on soft drinks are not identical. The baseline study estimated the share of calorie-reduced soft drinks in a sub-question, sports and energy drinks were asked separately. In KiGGS Wave 2, however, the consumption of calorie-reduced soft drinks was assessed in a separate question. Figure 1 shows the results of the conversion and aggregation of estimated daily intake of sugary soft drinks, calorie-reduced soft drinks and energy drinks in the KiGGS baseline study and Figure 2 for KiGGS Wave 2. These figures confirm the decrease in the levels of consumption, indicating that the development of consumption can only minimally be contributed to differences in the questions. However, changes in social desirability norms regarding the consumption of sugary soft drinks may have led to biased answers and some underreporting of consumption.

The observed change may be considered against the backdrop of preventive measures, such as improving the availability and attractiveness of drinking water at schools and kindergartens as an alternative to sugary soft drinks [18, 19]. In spite of the reported decrease, the consumption of sugary soft drinks remains high. Different prevention measures which may further reduce consumption are currently being discussed. Possible measures include a tax on sugary soft drinks, as well as stricter regulations for sugary soft drink advertisements directed at children and adolescents. Furthermore, it would be desirable to further extend the offer of unsweetened drinks (such as water, unsweetened teas) in nurseries, kindergartens and schools [1].

## Key statements

- Around 16.9% of girls and 22.2% of boys drink at least once a day sugary soft drinks.
- Consumption frequency of sugary soft drinks rises with age.
- Children and adolescents with low socioeconomic status consume more often sugary soft drinks compared to those of the same age with high status.
- The share of adolescents who drink sugary soft drinks daily has decreased since the KiGGS baseline study (2003-2006).

## Figures and Tables

**Table 1:** Prevalence of sugary soft drink consumption |according to gender, age and socioeconomic status (n=6,539 girls, n=6,439 boys) Source: KiGGS Wave 2 (2014-2017)

Less than once per day			One to three times per day		Four or more times per day	
	%	(95% CI)	%	(95% CI)	%	(95% CI)
**Girls (total)**	**83.1**	**(81.5-84.6)**	**13.7**	**(12.4-15.0)**	**3.3**	**(2.6-4.1)**
**Age**						
3-6 Years	90.5	(88.2-92.4)	8.1	(6.4-10.1)	1.4	(0.8-2.6)
7-10 Years	83.3	(80.8-85.6)	14.5	(12.4-16.8)	2.2	(1.5-3.3)
11-13 Years	79.0	(75.6-82.0)	15.6	(13.1-18.4)	5.5	(3.8-7.8)
14-17 Years	78.9	(75.8-81.6)	16.7	(14.4-19.3)	4.4	(3.1-6.3)
**Socioeconomic status**						
Low	74.8	(70.8-78.4)	19.8	(16.7-23.3)	5.4	(3.7-7.8)
Medium	82.1	(80.2-83.9)	14.4	(12.9-16.0)	3.5	(2.6-4.6)
High	95.0	(93.6-96.0)	4.6	(3.6-5.9)	0.4	(0.2-0.8)
**Boys (total)**	**77.8**	**(76.2-79.3)**	**17.6**	**(16.3-18.9)**	**4.7**	**(3.9-5.5)**
**Age**						
3-6 Years	87.1	(84.4-89.4)	10.3	(8.2-12.8)	2.6	(1.5-4.5)
7-10 Years	81.1	(77.9-83.9)	15.3	(13.0-18.1)	3.6	(2.5-5.2)
11-13 Years	74.8	(71.6-77.8)	19.8	(17.0-22.9)	5.4	(3.8-7.6)
14-17 Years	67.8	(64.6-70.9)	25.1	(22.3-28.1)	7.1	(5.6-9.0)
**Socioeconomic status**						
Low	64.5	(59.8-68.9)	25.6	(21.5-30.2)	9.9	(7.3-13.2)
Medium	77.5	(75.6-79.3)	18.2	(16.5-20.0)	4.3	(3.5-5.4)
High	91.1	(89.2-92.7)	8.0	(6.5-9.8)	0.8	(0.4-1.6)
**Total (girls and boys)**	**80.4**	**(79.1-81.6)**	**15.7**	**(14.7-16.7)**	**4.0**	**(3.5-4.6)**

CI=confidence interval
